# Factors affecting patient decisions to undergo testing for cancer symptoms: an exploratory qualitative study in Australian general practice

**DOI:** 10.3399/BJGPO.2022.0168

**Published:** 2023-02-08

**Authors:** Brent Venning, Rebecca Bergin, Alison Pearce, Alex Lee, Jon D. Emery

**Affiliations:** 1 Department of General Practice, Melbourne Medical School, University of Melbourne, Melbourne, Australia; 2 Centre for Cancer Research, University of Melbourne, Melbourne, Australia; 3 Cancer Epidemiology Division, Cancer Council Victoria, Melbourne, Australia; 4 Daffodil Centre, The University of Sydney, a joint venture with Cancer Council NSW, Sydney, Australia; 5 Sydney School of Public Health, The University of Sydney, Sydney, Australia

**Keywords:** general practice, cancer symptoms, investigations, qualitative research

## Abstract

**Background:**

Patients presenting to their GP are often concerned their symptoms may be due to cancer. However, there is a lack of evidence on the factors that influence patient decisions to undergo investigation for suspected cancer in the general practice setting.

**Aim:**

To identify the factors influencing patient decisions to undertake investigations for suspected cancer in general practice.

**Design & setting:**

An exploratory qualitative, semi-structured interview study of patients attending rural and metropolitan general practices in Victoria, Australia.

**Method:**

A purposive sample of 15 general practice patients aged ≥40 years participated. Thematic analysis of transcripts drew on interpretative description methodology and shared decision-making (SDM) theory.

**Results:**

Cancer-related concerns such as ‘cancer worry’ prompt patients to seek investigations from their GP. Participants prefer that their symptoms are investigated regardless of cancer risk. The perceived ‘best test’ provides the most reassurance. Trust and SDM enhance dialogue between patients and GPs about diagnostic testing strategies. Deterrents to testing included out-of-pocket costs, waiting time, travel time, and competing work and family demands.

**Conclusion:**

There may be a mismatch between efforts to rationalise investigation use and patient preferences for investigation. SDM that incorporates patient concerns, facilitators, and barriers to testing may ensure appropriate and timely investigation of cancer symptoms.

## How this fits in

Existing research focuses on the factors associated with the delayed presentation of symptomatic patients to general practice or delays in accessing treatment. There has been less emphasis on the variables influencing patient decisions to undergo initial diagnostic testing. This study highlights how factors such as ‘cancer worry’ motivate patients to request investigations and how barriers, such as cost and inconvenience, may result in testing delays. Incorporating these factors into shared decisions about testing strategies may result in improved dialogue and SDM between patients and GPs.

## Introduction

Patients often present to general practice with undifferentiated symptoms. These symptoms are more commonly owing to benign conditions, but can also represent less common but serious conditions such as cancer. In fact, most cancers are diagnosed after a symptomatic presentation to general practice, including cancers where screening programmes exist.^1,2^ Differentiating which patients need further investigation can be challenging in its own right, but this also needs to be balanced with patient preferences while avoiding unnecessary or wasteful testing.

The Aarhus statement to improve the design and reporting of studies on early cancer diagnosis defines the time points and intervals leading to cancer diagnosis and treatment.^3^ This includes the point at which patients first recognise symptoms and decide to discuss these with a clinician (appraisal interval), followed by the point at which the first consultation is performed (help-seeking interval), a diagnosis is made (diagnostic interval), and then treatment is started (pre-treatment interval).^3,4^ This article focuses on the decision-making process around the first investigations initiated in primary care, which forms the initial part of the diagnostic interval.

Extensive literature has examined the factors associated with symptom appraisal and help-seeking intervals.^5–9^ Less is known about the factors that patients consider when making decisions about different testing strategies with their GP. A vignette study by Banks *et al* highlighted a preference for cancer investigations across a range of symptom risks. Investigation choice was influenced by the invasiveness of a test (for example, colonoscopy) and not by the cancer type or treatment options.^10^ Other evidence suggests that patients place a greater value on the perceived quality of an investigation than the experiences related to the test such as discomfort.^10,11^ Evidence on preferences for cancer screening tests in asymptomatic patients has identified a range of other factors that influence patient decisions, including not only test-specific elements (for example, test procedure, accuracy) but also aspects of service delivery (for example, waiting time, location), potential outcomes (for example, adverse events), and cost.^12^ There is a lack of evidence about how a combination of these factors is incorporated into symptomatic testing decisions.

Patient preferences to investigate low-risk cancer symptoms has significant consequences for resource allocation and cost-effective testing. Concerns about the overuse of medical tests have been the focus of awareness campaigns such as Choosing Wisely, both in Australia and internationally, to shift practice away from what has been coined ‘low-value’ tests.^13^ This is especially pertinent in Australia, where GPs have greater direct access to diagnostic tests compared with other high-income nations such as the UK or Denmark.^14^ Greater flexibility of this ‘gatekeeping’ function has been suggested to contribute to better cancer outcomes in Australia.^14,15^ However, increased access to investigations also raises concerns about overtesting, particularly for low-risk scenarios that may be better managed with alternative, more efficient diagnostic strategies (for example, symptom monitoring and safety-netting).

Simultaneously satisfying patient preferences and avoiding over-investigation is a challenge that may be better understood through the lens of SDM.^16,17^ SDM models are incorporated into clinical guidelines to provide a framework for joint decision making between patients and clinicians, and are ideally suited to scenarios where there is a mismatch between GP and patient perspectives about testing.

The overarching purpose of this exploratory qualitative study was to identify attributes for a future discrete choice experiment (DCE) on patient preferences for cancer investigations.^18,19^ DCEs are a quantitative method for eliciting preferences by asking participants to state their preferred choice across competing scenarios, each consisting of a combination of different characteristics, termed attributes.^20^ This study therefore aimed to explore the factors that influence patient decisions to undergo testing when consulting their GP about symptoms that may be due to undiagnosed cancer.

## Method

The conduct and reporting of this study were guided by the Standards for Reporting Qualitative Research (SRQR).^21^

### The research team and reflexivity

BV, a male GP and PhD candidate, conducted the participant interviews. Participants were informed that the interviewer was a GP, and care was taken to outline BV's role in the interviews as a researcher only. None of the participants interviewed knew BV before the study. The broader research team consisted of one academic GP and three experienced non-clinician researchers with expertise in qualitative research design, SDM, and oncology research. Clinician–researchers have a unique understanding of patient issues, which helps inform the research’s practical implications. However, this can also introduce a power imbalance that may influence participant responses.^22,23^ The involvement of non-clinician researchers in data analysis and interpretation helps to counteract biases and assumptions introduced by clinician–researchers.^22^

### Study design

#### Theoretical framework

This qualitative study drew on interpretative description (ID) methodology. While initially developed to address the needs of nurse researchers, ID is well suited to examining clinical situations in other applied health disciplines, such as general practice, which seek to understand patients' subjective experiences and use this to inform clinical practice.^24^ ID recognises the interactions between psychosocial and biological phenomena and employs systematic reasoning to orient research towards the clinical context.^24,25^ SDM theory provides the theoretical scaffolding for data analysis, and encourages clinicians and patients to openly discuss advantages, disadvantages, and alternative options for diagnosis or treatment while also considering patient values and preferences before arriving at a final decision.^16^ Importantly, this means considering the breadth of factors that inform patients’ decisions, including not only clinical factors (for example, test risks or efficiency) and disease factors (for example, symptom severity, fear) but also psychological (for example, perception of risk), social (for example, life events, family), relationship (for example, relationship with a clinician), and health system factors (for example, access, waiting times).^16^

#### Study setting and participant selection

A purposeful sample of patients aged ≥40 years from rural and metropolitan general practices in Victoria was selected. Recruitment was undertaken in three ways. First, a poster advertising the study was placed at the reception and in the waiting room of participating general practices. Second, participants were identified from daily patient bookings and invited to join in person or via telephone. Third, participants from other studies within the Department of General Practice at The University of Melbourne who provided consent to be contacted for further research were selectively invited to participate via email.

There was no requirement for participants to be consulting a GP for investigations at the time of recruitment. However, it was expected that many patients in a GP waiting room would have experienced seeing a GP about symptoms of concern and discussed investigation options. An age threshold of 40 years was used for two reasons. First, approximately 20% of cancers occur in the 40–59 age group, and the predicted rate of new cancer cases over the next decade is greatest for people aged 40–49 years.^26^ Second, similar UK studies have focused on patients aged ≥40 years, enabling comparison with those findings.^10,27^ Exclusion criteria were: no proficiency in English, and no competency to consent for the interview.

A $50 AUD (approximately £30) gift card was used as an incentive for participation. In addition, participants were given an information sheet about the study and written informed consent was obtained before the interviews.

#### Data collection

A brief sociodemographic questionnaire was developed to understand the characteristics of the participants (*see appendix S1*). The interview guide addressed the research question by drawing on the author’s clinical experience and the existing literature. The interview guide enquired about participants' experiences attending their GP with symptoms what conditions often caused them concern and why . They were asked about their experiences with the investigative process, variables that influenced their decision to undergo testing, and how decisions regarding testing were influenced when cancer may be suspected (*see appendix S1*). The Primary Care Collaborative Cancer Clinical Trials Group (PC4) community advisory committee members contributed written feedback on the initial interview guide. A simulated interview was used to test the interview guide. To improve clarity, minor changes were made to the question structure and terminology.

The interviews were undertaken between 9 May and 1 July 2022. Interviews were conducted in person in a quiet room within the practice or via videoconference, depending on participant preference. Interviews lasted an average of 24 minutes (range 19–31 minutes). Interviews were audio-recorded, transcribed verbatim, and de-identified for analysis.

#### Data analysis

While an inductive thematic analysis was conducted, there was an element of deductive analysis due to the focus of the research question and the purpose of the study. The coding process drew on Braun and Clarke’s six phases of thematic analysis and recommendations regarding qualitative methods for attribute development for DCEs.^19,28^ The transcripts were loaded into NVivo software (release 1.0, released in March 2020) for line-by-line coding.^29^ Both BV and RB read an initial set of three transcripts. An initial set of candidate themes were created, and a thematic map was generated. Starting preliminary data analysis after several interviews assisted in refining thematic categories, enabling these to be explored further in subsequent interviews. Collated excerpts for each theme were examined and discussed to ensure consistency and coherence. The candidate themes and map were discussed with the research team during fortnightly meetings. At each meeting, the codes were revised until a final set of themes was agreed on.

## Results

### Participant characteristics

Twenty-five people were invited to participate, and 15 agreed to be interviewed for the study. This sample size was sufficient to explore the specific focus of the research study and its primary purpose to facilitate attribute development for a DCE. Participant demographic characteristics are outlined in Table 1.

**Table 1.** Demographic characteristics of participants (*n* = 15)

Demographic characteristic	*n*	%
Age, years		
40–49	1	6.7
50–59	4	26.7
60–69	8	53.3
≥70	2	13.3
Sex		
Female	10	66.7
Male	5	33.3
Location		
Rural	9	60.0
Metropolitan	6	40.0
Educational attainment		
High school	7	46.7
Certificate or diploma	6	40.0
University degree	2	13.3
Employment status		
Casual	1	6.7
Part-time	6	40.0
Full-time	2	13.3
Retired	5	33.3
Unemployed	1	6.7
Private health insurance		
Yes	8	53.3
No	7	46.7

### Interview findings

Six key themes were developed through data analysis, summarised in Table 2 and outlined in detail below with illustrative quotations.

**Table 2.** Themes and sub-themes derived from interviews with 15 Australian GP patients

Theme	Sub-theme
Theme 1: Cancer concerns that impact testing preferences	Motivated by cancer worrySeeking early detectionTesting provides reassurance
Theme 2: Preferences for different approaches to testing	Less invasive initial tests are acceptableThe best test is always preferred
Theme 3: Test specific factors patients discuss with their GP	Elements of the testing processesRisks associated with different tests
Theme 4: Social and health system influences on test choice	Waiting time for the test and test resultsTravel time to have the diagnostic testBalancing work and family demands
Theme 5: The cost of testing	Out-of-pocket costsPrivate versus public system
Theme 6: Aspects of the patient–GP relationship that influence testing	Clinician trustShared decision making

#### Theme 1: Cancer concerns that impact testing preferences

Participants described concerns that facilitated timely diagnostic testing. First, participants expressed ‘worry’ that the symptoms they had experienced may be owing to cancer. For some participants, this could be pervasive and only relieved by the reassurance provided through further investigation. This concern was amplified when participants had a previous cancer diagnosis personally or in a family member. As one participant described:

*'I mean, it is just so natural that having dealt with cancer so much in my life, with everything, I think, “okay, is this cancer?” All the time, “okay, is this cancer?”*' (ID 13, female, aged 62 years, metropolitan)

Participants reported seeking investigations even when they were aware their symptoms had a low possibility of being due to cancer. Some participants reported a sense of uncertainty when no testing was performed after raising concerns with their GP. The low probability that a symptom was caused by cancer was not regarded as reassuring. As one participant stated:

*'It wouldn’t matter what the symptom was, one per cent or five per cent. I’d still want it done because my fear of getting cancer outweighs the percentage. I want to know I’m fine.*' (ID 11, female, aged 64 years, metropolitan)

A subset of participants were motivated to pursue investigations through the desire for early detection and intervention, believing that doing so would enhance prognosis and future quality of life. One participant stated:

*'Having the tests, if it identifies something that can be nipped in the bud, fixed up, whatever. You ultimately end up with a better quality of life.*' (ID 10, male, aged 59 years, metropolitan)

#### Theme 2: Preferences for different approaches to testing

Participants preferred testing strategies that provided the most reassurance. Invasive diagnostic tests, such as colonoscopy, were perceived as more thorough than alternative tests for lower gastrointestinal symptoms such as faecal occult blood testing. These were often preferred despite increased risks:

*'Colonoscopy, I’d probably prefer to know for sure. I prefer the certainty, yeah, because I would think if I had that sample, I’d hope it’s accurate sort of thing.*' (ID 7, female, aged 59 years, rural)

The desire for more thorough testing strategies was often rooted in the desire for reassurance (see theme 1). It was sometimes driven by patient concerns rather than those of their GP:

*'The ovarian cancer stuff, that was very much initiated by me … I used to have a lot more concern around that than I probably do these days, but I was always upfront with saying I feel a bit concerned about that, so I want to be more thorough.'* (ID 14, female, aged 61 years, rural)

Participants perceived GPs who undertook further investigations as being more thorough. In addition, investigations provided reassurance and confidence that all potential paths for determining the cause of their symptoms were being pursued:

*'I probably would prefer to have an extra test rather than risk missing something. Because I think then I’ve done all I can, the doctor has done all they can, and the rest is how it is.'* (ID 09, female, aged 62 years, rural)

Nevertheless, some participants were open to a stepwise approach to diagnostic testing where appropriate, starting with initial less invasive tests and progressing to further testing if clinically indicated:

*'I would always look for the easier, quicker, simpler option. Yeah, and I would be quite happy with the answers that they gave me if they were okay. If something were recommended from that, to go further, I would deal with that, but I would always go the easier option.'* (ID 3, female, rural, aged 53 years, rural)

#### Theme 3: Test specific factors patients discuss with their GP

Select participants had concerns about the testing process, such as claustrophobia for computerised tomography (CT) or magnetic resonance imaging (MRI) scans, or bowel preparation for colonoscopy, although these were rarely considered barriers to testing:

*'Well, I actually hate having a colonoscopy. That stuff I have to take … it makes me vomit. It’s really bad. But it doesn’t deter me from the test, because I’d rather have that than miss a cancer.*' (ID 11, female, aged 64 years, metropolitan)

Other participants considered the risks associated with tests, such as pain or phobias related to blood tests, radiation for imaging modalities, risks associated with procedural sedation, and complications of procedures such as bowel perforation for colonoscopy:

*'There was concern about having too many scans and things like that. I don’t fully understand it … it’s not chemical, that’s not the word, but what they use in those X-rays.'* (ID 09, female, aged 62 years, rural)

Despite reporting awareness of risks, participants viewed the necessity of an investigation to outweigh any associated risks:

*'To be quite honest, I never worried about the risk. I know there’s a risk for any medical procedure, but I put the benefits way higher than the risks. So, the risks to me don’t come into it.*' (ID 4, female, aged 67 years, rural)

#### Theme 4: Social and health system influences on test choice

Participants identified health system factors, such as longer wait and travel times, as issues they discuss with their GP when deciding on testing options. Waiting time was significant for participants who used the public rather than the private system. While this related to waiting time for the test, it also included waiting time between test requests and follow-up appointments to discuss investigation results:

*'Over a few weeks that I had to wait in between appointments, the fear was pretty consuming at the time, yeah. Yeah, it all turned out to be all right, but it was just the process.'* (ID 2, female, aged 55 years, rural)

Participants reported a trade-off between waiting time and symptom severity. Some participants sought options in the private health system if their symptoms were concerning, which would expedite the process. As one participant put it:

*'If I hadn’t been dealt with quickly enough, or I wasn’t going to be dealt with quickly enough, for my wellbeing, then yeah, I would certainly go looking elsewhere.'* (ID 3, female, aged 53 years, rural)

Participants reported the dilemma posed by limited access to specific tests such as MRI scans. Where access to local services was limited, travel time to appointments was frustrating and a barrier for participants living in rural areas. Others acknowledged this as an inconvenience but not a barrier to testing, prioritising the need for tests over the additional travel:

*'It wouldn’t worry me if I had to drive if it was important for something that was a worry.*' (ID 7, female, aged 59 years, rural)

For those working full-time or with young families, there were often challenges in arranging times to attend appointments, and this was a potential cause for delay:

*'I wouldn’t have said I’d put one off. Well, you probably do, you’ve got to work it around your family a little bit, but I wouldn't put it off for months at a time.'* (ID 1, female, aged 45 years, rural)

#### Theme 5: The cost of testing

The cost of medical tests was an important consideration for some participants. Participants with financial difficulties would look for specific services with no out-of-pocket costs, either by asking for a referral to a bulk-billing service or shopping around until one was found. However, for some participants, out-of-pocket costs for testing would result in delays, as one person recounted:

*'I’ve had to give myself an extra week or two to know I’ve got the money to pay for it. That’s happened a couple of times.*' (ID 3, female, aged 53 years, rural)

However, other participants reported that even when the cost was a barrier, their medical needs would take priority, often drawing on various motivators for testing (theme 1), such as early detection or reassurance:

*'Money sometimes comes into how I’m feeling — I think, “oh gee, it’s a lot of money.” But then I think to myself, it’s better that I pay it and know that I’m clear.'* (ID 11, female, aged 64 years, metropolitan)

#### Theme 6: Aspects of the patient–GP relationship that influence testing

A strong patient–GP relationship was an important factor underpinning participant confidence in investigative strategies. Fundamental to this was trust. Participants reported establishing trust through an existing relationship with a regular GP, a GP’s inherent role as a trusted medical expert, or a consulting style that reassured participants. As one participant stated:

*'He was quite nice, and sort of showed that he cares about his patients. So, therefore, I trusted him in his judgement and whatever he was doing.'* (ID 13, female, aged 62 years, metropolitan)

When participants trusted their doctor, they were confident regarding proposed diagnostic strategies. In contrast, where there was a lack of established trust, participants showed increased uncertainty about a recommendation, particularly when there was a mismatch between their expectations for testing and GP recommendations. However, high levels of trust could also result in participants adopting a passive role in decision making:

*'I probably haven't asked too many questions about it. I suppose I'm very trusting in the fact that I'm being guided by professionals who are doing the right thing by me.'* (ID 01, female, aged 45 years, rural)

However, regardless of trust, other participants expected to be actively engaged in discussions about testing strategies to understand the indications for investigations.

*'I would need to understand why I’d be having the test, regardless of who the doctor was. So, I wouldn’t just go and do tests without explanation as to why they were doing it … As long as there’s a valid reason, I would do it.'* (ID 09, female, aged 62 years, rural)

## Discussion

### Summary

Incorporating patient preferences into clinical decisions is a fundamental aspect of patient-centred care. This study has highlighted how cancer-related concerns, such as ‘cancer worry’, are powerful motivators for patients to pursue diagnostic testing even for low-risk symptoms. Participants sought reassurance and expressed reservations about conservative testing strategies, such as 'watchful waiting', preferring earlier higher-order tests such as CT scans or diagnostic procedures. Few participants were concerned with the risks of investigations. Additional influences on people’s decision making included system factors, such as waiting time, travel time, and out-of-pocket costs, as well as competing work and family demands. There may be a mismatch between efforts to rationalise investigation use and patient preferences for investigation. SDM, underpinned by guidelines and health service structures that reflect patient concerns, may ensure appropriate, efficient, and timely investigation of cancer symptoms.

### Strengths and limitations

The research has allowed for in-depth analysis and comparison of the factors influencing patient decisions about primary care investigations. Techniques were employed to improve study rigour, including the following: having two people code a series of transcripts; iteratively discussing themes; moving back and forth between the thematic map and the transcripts to ensure that the developing coding scheme reflected the data; and having regular meetings at which the broader research team discussed the developing coding framework. Recruitment was from Victoria’s rural and metropolitan areas, improving the sample’s sociodemographic diversity. However, the sample was overrepresented by rural and female participants and limited to English-speaking participants. Data were collected across only two rural general practices in Victoria, meaning some findings may relate to issues faced by patients in these specific practices and communities. Compared with other states in Australia, rural populations in Victoria live comparatively closer to metropolitan centres, meaning the experiences of the rural participants may differ from patients in more sparsely populated regions of Australia.

### Comparison with existing literature

The findings make an important contribution to the literature. While much research has focused on the factors associated with delays in patients presenting to general practice (that is, symptom appraisal and help-seeking),^5–31,30,31^ a limited amount of research has focused on the factors considered by patients when making decisions with their GP on preferred diagnostic strategies to investigate cancer symptoms.

Participants in the study expressed concern that minor symptoms could be caused by cancer and sought early detection or reassurance through testing. This is an important factor to consider when understanding patient preferences for testing, particularly in the setting of overtesting, where the risk of cancer is low. A vignette study by Banks *et al* highlighted that patients wanted to be investigated for symptoms across both low and high cancer risk levels.^10^ This is relevant to patient preferences for testing as the presence of cancer worry among the general population is relatively common. A UK study found that the majority of English adults worry at least occasionally about developing cancer.^32^ In a recent Danish survey, half of patients reported worrying ‘a little about cancer’, and 37% ‘a lot about cancer’ when consulting their GP.^33^ This has clinical implications as misalignment between patient concern and GP suspicion increases the likelihood of having a prolonged time to diagnosis.^33^

The study found that GP trust and involvement in decision-making about investigations influenced patient confidence in the diagnostic process. Trust in doctors was one of four criteria conceptualised in a prior synthesis of the qualitative research on the determinants underlying effective patient—doctor relationships.^34^ While other aspects of a patient—doctor relationship, such as continuity of care, were influential in other studies, that was less relevant to the participants in the present study.^35^ Reasons for this may relate to the structure of the Australian primary care system, which allows patients to choose and move between different GPs and practices.

Participants in the study regarded clinicians who ordered investigations to be more thorough. Many patients will likely attend their GP expecting to be referred for investigations. For instance, a waiting-room survey of general practice patients found that more than one-quarter of patients wanted blood tests before seeing their GP.^36^ Expectations for investigations may have implications on the testing patterns of GPs, with focus group research highlighting the effect of perceived pressure on decisions to investigate.^37^ Most patients found conservative strategies, such as watchful waiting, less acceptable than pursuing definitive diagnostic testing, even if the test carried risks that may outweigh the benefits. How safety-netting strategies are discussed with patients impacts their acceptability.^38^ Passive rather than active safety-netting can be interpreted as being dismissive and may create a greater sense of uncertainty for patients.^38^ When patients are looking for reassurance through investigations, improved dialogue about different diagnostic strategies, such as watchful waiting, might help to address people’s concerns and enhance the acceptability of these options.

The study found that travel time, waiting time, and the cost of investigations were incorporated into patient decision making about diagnostic testing strategies. Limited access to diagnostic tests, such as CT scans, and specialised tests, such as colonoscopies, contribute to diagnostic delays in rural populations.^31,39^ Rural patients balance travel time to appointments against other work and family commitments. In such instances, local testing options may be preferred over other options located further away, even if they have greater diagnostic accuracy. Waiting times for procedures, such as diagnostic endoscopy, are considerably shorter in the private system. This is important as three-quarters of diagnostic colonoscopies in Australia are performed privately, while 55% of Australians have no private hospital insurance.^40,41^ Therefore, many patients need to weigh up the anxiety associated with diagnostic uncertainty and the waiting time for the tests against the cost of paying to have diagnostic procedures sooner through the private system.^40^

High out-of-pocket fees are a financial barrier to accessing specific diagnostic tests for some patients. Out-of-pocket costs for consumers account for 17% of healthcare spending in Australia, which is considerably higher than in other countries with similarly structured health systems.^42^ While out-of-pocket costs for pathology services have remained stable, diagnostic imaging contributes the fourth-highest out-of-hospital out-of-pocket costs for patients, with approximately 70% spent on ultrasounds.^42^ Rising out-of-pocket costs for investigations may be an obstacle to timely investigation and diagnosis for cancer, particularly for lower socioeconomic groups, creating inequities in outcomes.

### Implications for research and practice

This study has highlighted various factors that could be incorporated into SDM about diagnostic testing strategies for cancer symptoms in general practice. By highlighting a mismatch between campaigns such as Choosing Wisely and patient preferences to be investigated at low thresholds using more intensive testing, the findings have implications for efforts to rationalise primary care investigations. In addition, the study has highlighted important implications for policymakers, emphasising the challenges posed by health system factors, such as travel time, waiting time, and the cost of investigations on the decisions of patients. While this study describes factors that influence patient decision making for diagnostic testing, it does not identify which factors patients consider most important, how these may be traded off in different scenarios, or how testing preferences vary across different subgroups of patients. Further research using a DCE could provide a more comprehensive picture of patient decision making. Research on this topic with non-English speaking people and other minority groups is also warranted.

## Data availability

The de-identified data supporting this study’s results are available on request from the corresponding author.

## Data

The de-identified data supporting this study's results are available on request from the corresponding author.

## References

[1] Elliss-Brookes L, McPhail S, Ives A (2012). Routes to diagnosis for cancer — determining the patient journey using multiple routine data sets. Br J Cancer.

[2] Emery JD, Shaw K, Williams B (2014). The role of primary care in early detection and follow-up of cancer. Nat Rev Clin Oncol.

[3] Weller D, Vedsted P, Rubin G (2012). The Aarhus statement: improving design and reporting of studies on early cancer diagnosis. Br J Cancer.

[4] Walter F, Webster A, Scott S, Emery J (2012). The Andersen Model of Total Patient Delay: a systematic review of its application in cancer diagnosis. J Health Serv Res Policy.

[5] Kummer S, Walter FM, Chilcot J (2019). Do cognitive heuristics underpin symptom appraisal for symptoms of cancer?: a secondary qualitative analysis across seven cancers. Psychooncology.

[6] Birt L, Hall N, Emery J (2014). Responding to symptoms suggestive of lung cancer: a qualitative interview study. BMJ Open Respir Res.

[7] Hall N, Birt L, Banks J (2015). Symptom appraisal and healthcare-seeking for symptoms suggestive of colorectal cancer: a qualitative study. BMJ Open.

[8] Whitaker KL, Macleod U, Winstanley K (2015). Help seeking for cancer “alarm” symptoms: a qualitative interview study of primary care patients in the UK. Br J Gen Pract.

[9] Whitaker KL, Ghanouni A, Zhou Y (2017). Patients’ preferences for GP consultation for perceived cancer risk in primary care: a discrete choice experiment. Br J Gen Pract.

[10] Banks J, Hollinghurst S, Bigwood L (2014). Preferences for cancer investigation: a vignette-based study of primary-care attendees. Lancet Oncol.

[11] von Wagner C, Halligan S, Atkin WS (2009). Choosing between CT colonography and colonoscopy in the diagnostic context: a qualitative study of influences on patient preferences. Health Expect.

[12] Hall R, Medina-Lara A, Hamilton W (2022). Attributes used for cancer screening discrete choice experiments: a systematic review. Patient.

[13] Born K, Kool T, Levinson W (2019). Reducing overuse in healthcare: advancing choosing wisely. BMJ.

[14] Lynch C, Harrison S, Emery JD (2022). Variation in suspected cancer referral pathways in primary care: comparative analysis across the International Benchmarking Cancer Partnership. Br J Gen Pract.

[15] Vedsted P, Olesen F (2011). Are the serious problems in cancer survival partly rooted in gatekeeper principles? An ecologic study. Br J Gen Pract.

[16] Hoffmann TC, Légaré F, Simmons MB (2014). Shared decision making: what do clinicians need to know and why should they bother?. Med J Aust.

[17] Elwyn G, Cochran N, Pignone M (2017). Shared decision making-the importance of diagnosing preferences. JAMA Intern Med.

[18] Bridges JFP, Hauber AB, Marshall D (2011). Conjoint analysis applications in health — a checklist: a report of the ISPOR Good Research Practices for Conjoint Analysis Task Force. Value Health.

[19] Coast J, Al-Janabi H, Sutton EJ (2012). Using qualitative methods for attribute development for discrete choice experiments: issues and recommendations. Health Econ.

[20] Lancsar E, Louviere J (2008). Conducting discrete choice experiments to inform healthcare decision making: a user’s guide. Pharmacoeconomics.

[21] O’Brien BC, Harris IB, Beckman TJ (2014). Standards for reporting qualitative research: a synthesis of recommendations. Acad Med.

[22] McNair R, Taft A, Hegarty K (2008). Using reflexivity to enhance in-depth interviewing skills for the clinician researcher. BMC Med Res Methodol.

[23] Richards H, Emslie C (2000). The “doctor” or the “girl from the university”? Considering the influence of professional roles on qualitative interviewing. Fam Pract.

[24] Thorne S (2016). Interpretive Description: Qualitative Research for Applied Practice.

[25] Hunt MR (2009). Strengths and challenges in the use of interpretive description: reflections arising from a study of the moral experience of health professionals in humanitarian work. Qual Health Res.

[26] Australian Institute of Health and Welfare (AIHW) (2021). Cancer in Australia. https://www.aihw.gov.au/reports/cancer/cancer-in-australia-2021/summary.

[27] Banks J, Walter FM, Hall N (2014). Decision making and referral from primary care for possible lung and colorectal cancer: a qualitative study of patients’ experiences. Br J Gen Pract.

[28] Braun V, Clarke V (2006). Using thematic analysis in psychology. Qual Res Psychol.

[29] NVivo (2020). Unlock insights with the leading qualitative data analysis software. https://www.qsrinternational.com/nvivo-qualitative-data-analysis-software/home.

[30] Macleod U, Mitchell ED, Burgess C (2009). Risk factors for delayed presentation and referral of symptomatic cancer: evidence for common cancers. Br J Cancer.

[31] Emery JD, Walter FM, Gray V (2013). Diagnosing cancer in the bush: a mixed-methods study of symptom appraisal and help-seeking behaviour in people with cancer from rural Western Australia. Fam Pract.

[32] Murphy PJ, Marlow LAV, Waller J, Vrinten C (2018). What is it about a cancer diagnosis that would worry people? A population-based survey of adults in England. BMC Cancer.

[33] Virgilsen LF, Pedersen AF, Vedsted P (2021). Alignment between the patient’s cancer worry and the GP’s cancer suspicion and the association with the interval between first symptom presentation and referral: a cross-sectional study in Denmark. BMC Fam Pract.

[34] Ridd M, Shaw A, Lewis G, Salisbury C (2009). The patient-doctor relationship: a synthesis of the qualitative literature on patients’ perspectives. Br J Gen Pract.

[35] Pandhi N, Bowers B, Chen F (2007). A comfortable relationship: a patient-derived dimension of ongoing care. Fam Med.

[36] van Bokhoven MA, Pleunis-van Empel MCH, Koch H (2006). Why do patients want to have their blood tested? A qualitative study of patient expectations in general practice. BMC Fam Pract.

[37] Opdal PØ, Meland E, Hjörleifsson S (2019). Dilemmas of medical overuse in general practice — a focus group study. Scand J Prim Health Care.

[38] Black GB, van Os S, Renzi C (2022). How does safety netting for lung cancer symptoms help patients to reconsult appropriately? A qualitative study. BMC Prim Care.

[39] Jordan SJ, Francis JE, Nelson AE (2010). Pathways to the diagnosis of epithelial ovarian cancer in Australia. Med J Aust.

[40] Emery JD, Kyriakides M, Faragher I (2021). Validation of Australian and Victorian guidelines for colonoscopy triage. Intern Med J.

[41] Australian Prudential Regulation Authority (APRA) Quarterly private health insurance statistics. https://www.apra.gov.au/quarterly-private-health-insurance-statistics.

[42] Australian Institute of Health and Welfare (AIHW) Pathology, imaging and other diagnostic services. https://www.aihw.gov.au/reports/australias-health/specialist-pathology-and-other-diagnostic-services.

